# Supplementary material for: A Comparative Study of Composition and Soluble Polysaccharide Content between Brewer’s Spent Yeast and Cultured Yeast Cells
Source: Foods. 2024 May 17;13(10):1567. doi: 10.3390/foods13101567 (PMC11121356; doi:10.3390/foods13101567)
Supplement: Supplementary file 1 [file foods-13-01567-s001.zip › foods-2991159-supplementary.pdf]

foods MDPI

Supplementary Data

Table S1. **Insoluble polysaccharide of alkali extraction derived from autolysate residue.**

Samples	β-glucan (as glucose)	Mannan/Mannoprotein (as mannose)	Chitin (as *N*-acetyl-glucosamine)
BSY	778.50 ± 20.25[a]	41.50±0.99[c]	3.61±0.11[c]
SC	631.86 ± 14.25[c]	61.78±1.63[a]	36.29±0.12[a]
SB	697.80 ± 10.13[b]	58.86±1.63[b]	23.13±0.15[b]

BSY, Brewer's spent yeast, SC: *S. cerevisiae*; SB: *S. boulardii*. Values are means ± SD (n = 3). [*]Means with different superscript in the same column are significantly different $p < 0.05$.
